# Structural Heart Issues in Dextrocardia: Situs Type Matters

**DOI:** 10.31486/toj.19.0119

**Published:** 2021

**Authors:** Charles J. Fox, Yenabi Keflemariam, Elyse M. Cornett, Richard D. Urman, Yury Rapoport, Bipin Shah, Mary C. Mancini, Alan D. Kaye

**Affiliations:** ^1^ Department of Anesthesiology, Louisiana State University Health–Shreveport, Shreveport, LA; ^2^ Department of Anesthesiology, Valley Baptist Medical Center–Harlingen, Harlingen, TX; ^3^ Department of Anesthesiology, Perioperative and Pain Medicine, Brigham and Women's Hospital, Boston, MA; ^4^ CHRISTUS Highland Medical Center, Shreveport, LA

**Keywords:** *Anesthesia*, *dextrocardia*, *echocardiography*, *heart septal defects—atrial*, *situs classification*

## Abstract

**Background:** Patients who are diagnosed with dextrocardia, a rare congenital heart condition in which the heart points toward the right side of the chest, need their specific situs classification (eg, solitus, inversus, ambiguus) ascertained to optimize their care and outcomes. In this report, we discuss the perioperative anesthetic management of a patient presenting with dextrocardia.

**Case Report:** A 44-year-old African American female with a history of hypertension, hyperlipidemia, gastroesophageal reflux disease, and diabetes mellitus type 2 was admitted for shortness of breath, dyspnea on exertion, orthopnea, and paroxysmal nocturnal dyspnea. The patient had been diagnosed with dextrocardia in 2003 at an outside hospital and was asymptomatic prior to this presentation. Chest x-ray revealed bilateral perihilar vascular congestion with bibasilar atelectasis and suspected small bilateral pleural effusions consistent with new-onset congestive heart failure. Preoperative 2-dimensional transthoracic echocardiography revealed an ostium secundum–type atrial septal defect (ASD) with mild left-to-right atrial shunting. The patient's ASD was repaired using a pericardial patch.

**Conclusion:** The anesthetic management of patients presenting with dextrocardia is complex. Preoperative cardiac transthoracic echocardiography can identify cardiac lesions or aberrant anatomy associated with dextrocardia. Proper placement of electrocardiogram electrodes is necessary to avoid false-positive results for perioperative ischemia. Central line access must be adjusted to anatomic variations. Clinicians should have high suspicion for associated pulmonary hypertension and should limit sedatives preoperatively to minimize the cardiovascular effects of hypoxia and/or hypercarbia on the pulmonary vasculature. Finally, high clinical suspicion for respiratory complications should be maintained, as dextrocardia has been associated with respiratory complications secondary to primary ciliary dyskinesia in approximately 25% of patients.

## INTRODUCTION

Dextrocardia is a congenital abnormality in which the heart is positioned in the right hemithorax. To classify the diagnosis, the situs type must be determined. The word *situs* refers to the arrangement of structures within the human body, and the 3 types are situs solitus, situs inversus, and situs ambiguus. In dextrocardia with situs solitus, the abdominal organs are in normal configuration, but the heart is located in the right hemithorax.^1^ The majority of patients with this condition have significant cardiac disorders; only 10% of patients with dextrocardia with situs solitus are free from significant cardiac pathology. In contrast, patients with dextrocardia with situs inversus—in which the abdominal organs are reversed or mirrored—have only a 5% to 10% chance of substantial cardiac pathology.^2^ Situs ambiguus is the most severe and disorganized visceral misalignment disorder among the dextrocardia variants.^3^ Patients with the condition are extremely complex anatomically, and one variant, situs ambiguus with polysplenia, has an extremely high mortality rate, with anomalies that include severe pulmonary hypertension.^4,5^

To ensure excellent clinical outcomes, clinicians must be aware of the patient's dextrocardia pathogenesis and use perioperative diagnostic tools such as x-ray, computed tomography (CT), echocardiography, and magnetic resonance imaging to confirm the dextrocardia diagnosis and classify the specific type of dextrocardia. Because dextrocardia with situs solitus primarily affects the cardiovascular system, quantifying the presence or absence of cardiac pathology is important. Even if patients have been previously diagnosed with dextrocardia and are asymptomatic, cardiac consultation for evaluation and a thorough medical history are necessary. The major concern for asymptomatic patients is the diagnosis of congenitally corrected transposition of the great arteries (ccTGA). If the diagnosis of ccTGA is made by echocardiography, further investigation is warranted to rule out increased ventricular end-diastolic pressures or intracardiac shunts.^6^ Patients who are pregnant should have abdominal imaging performed prior to delivery to rule out situs inversus secondary to aortocaval compression mismanagement.^7^ Aortocaval compression is a common occurrence during pregnancy, and delineation of anatomy will help define positioning or uterine displacement to avoid or treat this phenomenon.

Dextrocardia alone rarely affects airway pathology, but when dextrocardia is diagnosed as part of situs inversus, the patient's chance of having primary ciliary dyskinesia or Kartagener syndrome is 25%.^8^ When caring for patients with Kartagener syndrome, pulmonary complications are a primary concern. These patients should have a pulmonary consultation prior to any scheduled surgery to ensure medical optimization. A perioperative treatment regimen that includes antibiotic prophylaxis, administration of bronchodilators with chest physiotherapy, postural drainage, and incentive spirometry may ameliorate pulmonary complications. Additionally, patients with Kartagener syndrome may have immunologic issues resulting from impaired motility of their neutrophils and renal abnormalities resulting from polycystic kidney disease and/or glomerulopathies.

In this report, we discuss the anesthetic management of a patient presenting with dextrocardia with situs solitus and emphasize the importance of dextrocardia subtype diagnosis.

## CASE REPORT

A 44-year-old African American female with a medical history of hypertension, hyperlipidemia, gastroesophageal reflux disease, and diabetes mellitus type 2 presented with a 2-month history of worsening shortness of breath, dyspnea on exertion, orthopnea, and paroxysmal nocturnal dyspnea. Her family history was significant for a mother who was diagnosed with an atrial septal defect (ASD) when she presented to the emergency department with congestive heart failure (CHF). Our patient had been previously diagnosed with dextrocardia with situs inversus in 2003 at an outside hospital, but per her report, she was asymptomatic prior to this presentation.

Chest x-ray revealed bilateral perihilar vascular congestion with bibasilar atelectasis and suspected small bilateral pleural effusions consistent with new-onset CHF. Preoperative 2-dimensional transthoracic echocardiography (TTE) revealed mild bilateral atrial enlargement, mild left ventricular dilatation with an ejection fraction of 45% to 50%, possible bicuspid aortic valve, and an ostium secundum–type ASD with mild left-to-right atrial shunting. Transesophageal echocardiography (TEE) revealed a pulmonary to systemic flow (Qp/Qs) ratio of 1.3:1 and confirmed a trileaflet aortic valve. Left heart catheterization ruled out any significant coronary arterial disease but revealed moderate pulmonary hypertension and a dilated right ventricle with a Qp/Qs ratio of 1.6:1. Given the patient's clinical presentation and her Qp/Qs ratio, the cardiothoracic surgery team decided to perform an ASD closure.

In the operating room, standard anesthetic monitors were applied. Electrocardiogram (ECG) electrode placement was reversed to account for dextrocardia. A right radial arterial catheter was placed, and standard cardiac intravenous induction was performed, titrating doses of midazolam 2 mg, sufentanil 50 μg, and propofol 140 mg. Neuromuscular blockade was performed with rocuronium 1 mg/kg. After induction, the patient was successfully intubated on one attempt with a 7.5-mm endotracheal tube via direct laryngoscopy using a Macintosh 3 laryngoscope blade. Maintenance of anesthesia was achieved using isoflurane (dose range of 0.3% to 0.7% isoflurane), sufentanil 1.5 μg/kg/h, rocuronium 50 mg, and midazolam 2 mg.

Central line access was achieved by placing a 9 French introducer. Entrance into the right atrium was acquired by placement into the left internal jugular vein under ultrasound guidance. After placement of the introducer, a triple-lumen catheter placed through the introducer provided monitoring of central venous pressures and venous access for vasoactive medication infusion.

**Figure 1.**
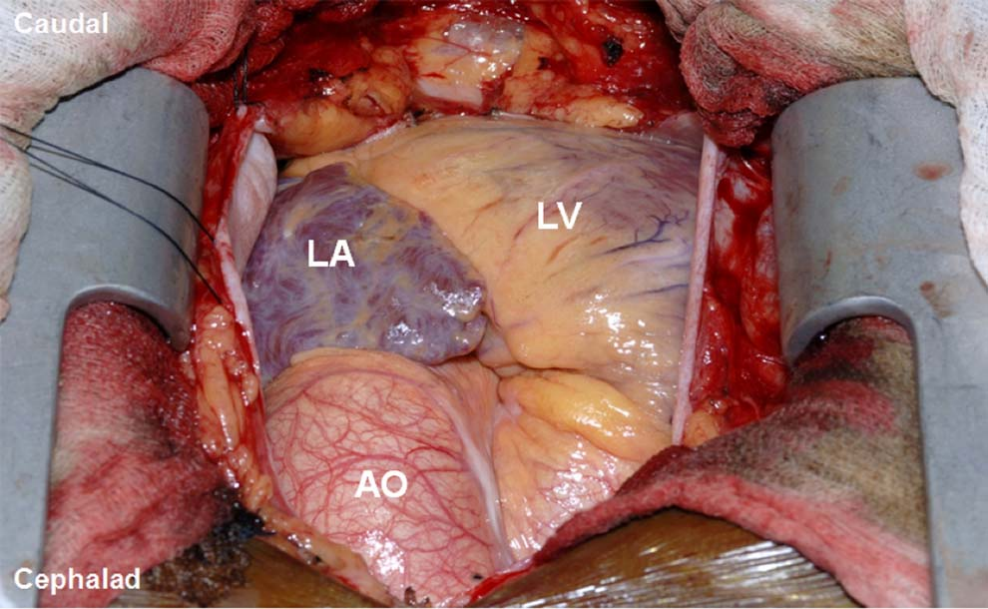
**Perioperative photograph shows the cardiac chamber anatomic variations in dextrocardia.** AO, aorta; LA, left atrium; LV, left ventricle.

**Figure 2.**
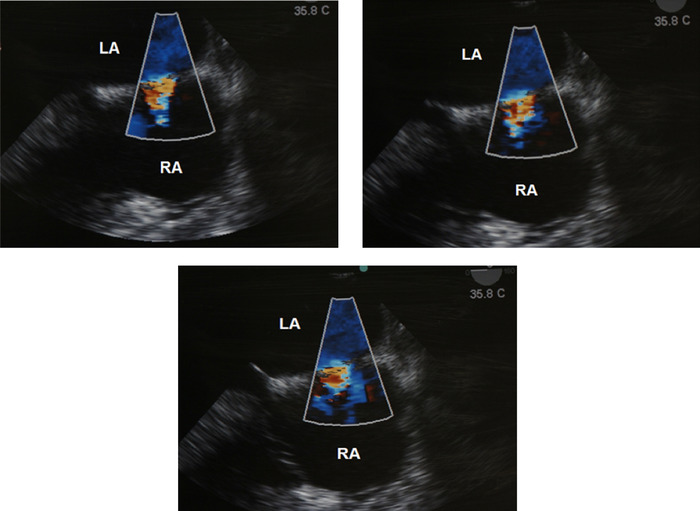
**Color-flow mapping Doppler images of transthoracic echocardiography show diastolic intra-atrial jet through the atrial septal defect. Flow toward the transducer is red, flow away from the transducer is blue, and higher velocities are shown in lighter shades. Turbulent flow is indicated by green.** (A color image is available online at https://doi.org/10.31486/toj.19.0119.) LA, left atrium; RA, right atrium.

Figure 1 shows the patient's cardiac chamber anatomic variations. Intraoperative TEE was used to appreciate the anatomy and to evaluate cardiac performance. In the midesophageal 4-chamber view, the ASD was identified and confirmed by the anesthesiology attending and cardiothoracic surgeon. Cardiac chambers and an intra-atrial jet through the ASD were observed via TTE (Figure 2).

**Figure 3.**
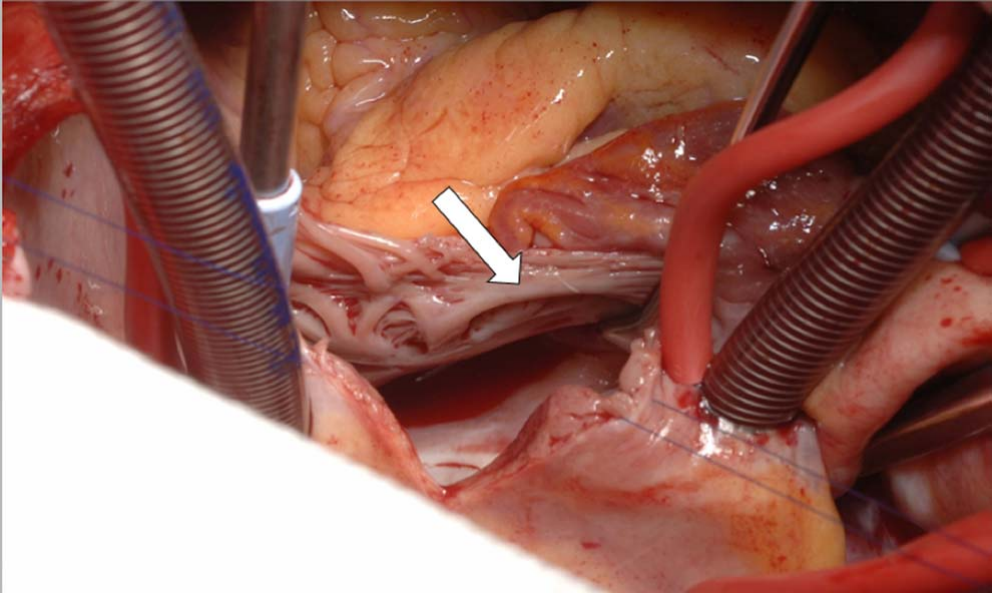
**Perioperative photograph shows a view of the patient's atrial septal defect through the right atrial window (arrow).**

The patient was then placed on cardiopulmonary bypass, and the cardiac surgeon closed the ASD (Figure 3) using a pericardial patch. After ASD closure and separation from cardiopulmonary bypass, no residual intra-atrial jet was seen. The patient was transported to the surgical intensive care unit and extubated via fast-track protocol by the cardiothoracic surgery team. The patient's hospital course was uneventful, and her symptoms of CHF resolved prior to discharge on postoperative day 4.

## DISCUSSION

The initial presentation of patients with dextrocardia can be challenging for the unsuspecting clinician. Providers must realize that the dextrocardia diagnosis is only the beginning of the diagnostic process; the specific situs classification must also be determined. The usual straightforward presentations of conditions such as acute appendicitis and cholecystitis, with their associated anatomic pain localizations, are reversed. The absence of heart sounds with auscultation can alert the clinician to a probable diagnosis of dextrocardia. Chest and abdominal radiography and CT imaging can confirm the presence of dextrocardia and situs classification.

In 2008, the American College of Cardiology/American Heart Association adult congenital heart disease guidelines stated that surgical or percutaneous closure of an ASD should be performed for patients with evidence of right atrial or ventricular enlargement with or without clinical symptoms.^6^ Qp/Qs ratios >2:1 are clear indications for ASD repair, yet some clinicians have moved toward repairing defects with ratios as low as 1.5:1.^6^ Historically, the gold standard for calculation of Qp/Qs ratio was heart catheterization, but according to a 2019 study, Doppler echocardiography can be used to evaluate pulmonary artery hypertension in some patients, particularly patients with mild pulmonary hypertension.^9^

## CONCLUSION

The anesthetic management of patients presenting with dextrocardia is complex, and this case elucidated several important points: (1) preoperative cardiac TTE or TEE can identify cardiac lesions or aberrant anatomy associated with dextrocardia; (2) proper placement of ECG electrodes is necessary to avoid false-positive results for perioperative ischemia; (3) central line access must be adjusted to anatomic variations; (4) given the clinical manifestations of right heart overload, clinicians should have high suspicion for associated pulmonary hypertension and should limit sedatives preoperatively to minimize the cardiovascular effects of hypoxia and/or hypercarbia on the pulmonary vasculature; and (5) high clinical suspicion for respiratory complications should be maintained because dextrocardia has been associated with respiratory complications secondary to primary ciliary dyskinesia in approximately 25% of patients.

## Notes

This article meets the Accreditation Council for Graduate Medical Education and the American Board of Medical Specialties Maintenance of Certification competencies for Patient Care and Medical Knowledge.
